# The biological function of the type II toxin-antitoxin system *ccdAB* in recurrent urinary tract infections

**DOI:** 10.3389/fmicb.2024.1379625

**Published:** 2024-04-16

**Authors:** He Zhang, Shuan Tao, Huimin Chen, Yewei Fang, Yao Xu, Luyan Chen, Fang Ma, Wei Liang

**Affiliations:** ^1^Department of Medical Laboratory, Bengbu Medical University, Bengbu, China; ^2^School of Medicine, Jiangsu University, Zhenjiang, China; ^3^Department of Clinical Laboratory, The First Affiliated Hospital of Ningbo University, Ningbo, China; ^4^School of Medicine, Ningbo University, Ningbo, China; ^5^Department of Blood Transfusion, The First Affiliated Hospital of Ningbo University, Ningbo, China

**Keywords:** recurrent urinary tract infection, toxin-antitoxin systems, *E. coli*, bacteria biofilm, *ccdAB*, persistent infection

## Abstract

Urinary tract infections (UTIs) represent a significant challenge in clinical practice, with recurrent forms (rUTIs) posing a continual threat to patient health. *Escherichia coli* (*E. coli*) is the primary culprit in a vast majority of UTIs, both community-acquired and hospital-acquired, underscoring its clinical importance. Among different mediators of pathogenesis, toxin-antitoxin (TA) systems are emerging as the most prominent. The type II TA system, prevalent in prokaryotes, emerges as a critical player in stress response, biofilm formation, and cell dormancy. *ccdAB*, the first identified type II TA module, is renowned for maintaining plasmid stability. This paper aims to unravel the physiological role of the *ccdAB* in rUTIs caused by *E. coli*, delving into bacterial characteristics crucial for understanding and managing this disease. We investigated UPEC-induced rUTIs, examining changes in type II TA distribution and number, phylogenetic distribution, and Multi-Locus Sequence Typing (MLST) using polymerase chain reaction (PCR). Furthermore, our findings revealed that the induction of *ccdB* expression in *E. coli* BL21 (DE3) inhibited bacterial growth, observed that the expression of both *ccdAB* and *ccdB* in *E. coli* BL21 (DE3) led to an increase in biofilm formation, and confirmed that *ccdAB* plays a role in the development of persistent bacteria in urinary tract infections. Our findings could pave the way for novel therapeutic approaches targeting these systems, potentially reducing the prevalence of rUTIs. Through this investigation, we hope to contribute significantly to the global effort to combat the persistent challenge of rUTIs.

## Introduction

Urinary tract infection (UTI) ranks among the most prevalent clinical infectious diseases globally, impacting an estimated 15.01 billion people annually (Harding and Ronald, 1994). and stands as the second most common infection after respiratory diseases (McLellan and Hunstad, 2016). Research indicates that approximately 50–70% of women and 5% of men are diagnosed with a UTI at least once in their lifetimes (Geerlings, 2016). In contrast, women face a higher incidence of UTIs, attributed to anatomical factors such as the proximity of the urethra to the perianal area, facilitating easier bacterial migration (Al-Badr and Al-Shaikh, 2013). The current reliance on antimicrobial therapy has not only shifted the distribution of UTI pathogens but also spurred the emergence of tolerant bacteria. This, combined with the influence of host behaviors, immune responses, and environmental factors, has significantly contributed to the rise of rUTI.

The pathogenesis of recurrent urinary tract infections (rUTI) is multifaceted, commonly attributed to inadequate treatment of initial infections, bacterial colonization of the urinary tract’s mucosal epithelium, and compromised immunity. Research in this field has primarily concentrated on three areas: (1) bacterial biofilm formation, identified as a critical factor in clinically refractory UTIs, biofilms are microcolonies formed by single or multiple bacterial species in response to environmental stimuli (Dai et al., 2021). Biofilms at infection sites, or those contaminated with biological materials, are notoriously difficult to eradicate, even with high doses of antibiotics. This resilience contributes significantly to recurrent infections (Sycz et al., 2022). Uropathogenic *E. coli* (UPEC), as suggested in animal models, is particularly susceptible to biofilm formation at the renal proximal tubule (Melican et al., 2011). (2) Intracellular bacterial community (IBC), IBCs form when UPEC adheres to and invades bladder epithelial cells, creating biofilm-like multicellular colonies (Hirakawa et al., 2019). Normally, bacterial invasion triggers an immune response leading to the expulsion of bacteria. However, IBCs can re-emerge from within host cells, invading new epithelial layers and persisting despite the presence of antibiotics. (3) Persistent bacteria, are slow-metabolizing, genetically identical subpopulations within bacterial colonies, exhibiting high resistance to antibiotics and environmental stresses. Persistent bacteria are a key factor in the failure of antibiotic treatments. Clonal populations of bacteria, although sensitive to specific antibiotics, almost invariably contain slow-growing or dormant cells resistant to these drugs (Levin and Rozen, 2006).

The toxin-antitoxin (TA) system, prevalent in the genomes and plasmids of prokaryotes, is a crucial regulatory mechanism comprising two fundamental components: stable toxins and unstable antitoxins. Toxins typically inhibit key bacterial growth processes like DNA replication and protein translation, while antitoxins can counteract these toxins. Based on the nature of antitoxin and the mode of interaction, TA systems have been broadly divided into eight types (I–VIII). In the type I TA system, antitoxins are antisense RNAs that hinder toxin mRNA, either promoting its degradation or blocking ribosome binding, thus preventing toxin synthesis (Yamaguchi et al., 2011). The type II system, the most abundant and extensively researched, involves protein-based toxins and antitoxins that form a complex inhibiting TA system transcription (Li et al., 2020). Type III TA systems feature sRNA antitoxins directly interacting with toxin proteins, while type IV systems have protein-based antitoxins that block toxin targets (Guo et al., 2016). In type V, ribonuclease antitoxins degrade toxin mRNA (Wang et al., 2012), and type VI involves antitoxin proteins promoting toxin hydrolysis (Aakre et al., 2013). Type VII and VIII systems, both featuring protein or sRNA components, inactivate toxins through translational modification or antisense binding, respectively (Choi et al., 2018; Yu et al., 2020; Li et al., 2021).

Among them, type II TA systems are the most abundant and widely studied, to date more than 37 TA systems have mostly been identified in *E. coli* K-12 MG1655 (Harms et al., 2018). It plays an important role in a variety of biological processes, such as plasmid maintenance (Sonika et al., 2023), phage inhibition (LeRoux and Laub, 2022), stress response (Qiu et al., 2022), virulence (Wood and Wood, 2016), biofilm formation (Ma et al., 2019), and generation of dormant and persistent cells (Rycroft et al., 2018). *ccdAB* is the first type II TA system discovered to maintain plasmid stability and consists of two genes, *ccdA* and *ccdB* (Vandervelde et al., 2017). The *ccdA* protein is readily degraded by Lon protease and acts as an antitoxin in the system. *ccdB* protein is a cytotoxin that is more stable than the *ccdA* protein. The toxin *ccdB* targets the GyrA subunit of the DNA-promoter enzyme, inhibiting DNA replication and causing DNA damage. By inhibiting the DNA-promoter enzyme from catalyzing the rejoining of DNA in the cycle, *ccdB* locks the enzyme on the DNA into what is known as the cleavage complex, which works in a manner very similar to that of the quinolone antibiotics (Gupta et al., 2017). The natural chromosomal *ccd* operon of *E. coli* strain O157 was found to be involved in drug resistance, preventing cell death under multiple antibiotic stress conditions by forming persistence (Gupta et al., 2017). Exploring the distribution and biological role of the type II TA system in recurrent urinary tract infection strains is clinically important. Therefore, we detected 15 pairs of type II TA systems in recurrent urinary tract infection strains by PCR reaction, investigated the clinical epidemiological features, and explored the role of *ccdAB* in recurrent urinary tract infection strains.

## Materials and methods

### Bacteria strains, plasmids, and growth conditions

Bacteria strains and plasmids used or constructed in this study are listed in Table 1. *E. coli* BL21 (DE3) was used to make chemically competent cells and strains for plasmid transformation experiments and propagation. *E. coli* has grown in Luria-Bertani (LB, 5 g yeast extract, 5 g NaCl, and 10 g tryptone per liter) broth or on LB agar (LB supplemented with 15 g agar per liter) plates at 37°C. For the expression studies, plasmid pET28a, known for its IPTG-inducible expression system, was cultured under similar conditions at 37°C. However, induction of gene expression was achieved by the addition of 1 mmoL/L isopropyl β-D-1-thiogalactopyranoside (IPTG). Furthermore, Kanamycin was added to the medium at a concentration of 50 mg/mL whenever necessary to ensure efficient plasmid maintenance.

**Table 1:** Bacteria strains and plasmids in this study.

Bacterial strains and plasmids	Description	Reference or source
Strains		
*E. coli* BL21(DE3)	F-, ompT, hsdSB (rB-mB-) gal, dcm araB: T7RNAP-tetA	Laboratory stock
BL21-pET28a-ccdAB	IPTG-inducible expression plasmid	This study
BL21-pET28a-ccdA	IPTG-inducible expression plasmid	This study
BL21-pET28a-ccdB	IPTG-inducible expression plasmid	This study
BL21-pET28a	IPTG-inducible expression plasmid	This study
Plasmids		
pET28a(+)	Kanamycin resistance, expression vector	Laboratory stock

### Sample collection

Isolates were collected from March to August 2023 in the Clinical Microbiology Laboratory of the First Affiliated Hospital of Ningbo University. We specifically focused on female inpatients for sample selection. rUTI was defined as three or more episodes of urinary tract infection, in the past year and this study was medically diagnosed with recurrent urinary tract infections. For two or more recurrences within 6 months (Jung and Brubaker, 2019). The strains selected for comparison, we also screened for non-recurrent urinary tract infection cases. These were identified as patients who, during two UTI episodes, had at least one negative mid-stream urine culture or a normal leukocyte count in routine urine tests. Additionally, only those who had not received treatment from external institutions were considered eligible for inclusion in the study. This study’s protocol received the approval of the Ethics Committee of the First Affiliated Hospital of Ningbo University.

### Identification and confirmation of *E. coli*

All strains were inoculated on MacConkey agar plates and incubated at 37°C for 24 h. A colony with typical *E. coli* morphology and size was selected for Gram staining to observe whether it was a negative strain. Then pure colonies were identified as *E. coli* using an automated bacterial identification system (VITEK 2 Compact, Biomerieux, France) (Infante et al., 2021).

### Detection of type II toxin-antitoxin systems

For this research, we obtained 88 strains of rUTI and UTI from the First Affiliated Hospital of Ningbo University. The isolates were taken out from the −80°C refrigerator and resuscitated, inoculated on blood plates, and cultured in 37°C incubators for 18 h. The boiling method was applied for the extraction of the genomic DNA of the bacterial isolates (Dallenne et al., 2010). Briefly, single colonies were picked and suspended in 100 μL of sterile ultrapure water and boiled at 95°C for 10 min, followed by centrifugation at 12,000 rpm for 10 min. The supernatant was transferred to a sterile tube and stored at −20°C for subsequent PCR experiments. We then amplified 15 pairs of toxin-antitoxin genes using polymerase chain reaction (PCR) with the primers listed in Table 2. The PCR reaction mixture consisted of 10 μL, including 5 μL of distilled water, 5 μL of reaction mixture reagent (Nanjing Novozymes Bio-technology Co., Nanjing, China), 1 μL of DNA product extracted by boiling, and 0.2 μL each of forward and reverse primers. We conducted the PCR reaction with pre-denaturation at 94°C for 5 min, followed by 30 amplification cycles and further extension at 72°C for 5 min. Each cycle involved a denaturation step at 94°C for 30 s and an annealing/amplification step at 55°C for 30 s, followed by extension at 72°C for 30 s. Finally, PCR products were subjected to electrophoresis and photography under ultraviolet trans-illumination.

**Table 2:** Primer sequences of TA loci.

TA locus	Direction	Primers 5′–3′	Product size
*hipAB*	Forward Reverse	GAACTCTCAATGACGCTATG TCTGTCGGTAATGAAGTCTT	1,312 bp
*prlFyhaV*	Forward Reverse	GCTGTACTGACCACTGAA AATACGGTATAGGCATCTGT	700 bp
*phd/doc*	Forward Reverse	CAATCCATTAACTTCCGTACC CTACTCCGCAGAACCATAC	599 bp
*yoeByefM*	Forward Reverse	CCTTATTACTCGTCAGAATGG GCAATGAGCAGTGAATCG	401 bp
*yafNO*	Forward Reverse	GCATCGAATTCTCGCTGAACGAA ACGCTTCTGCCATT	680 bp
*vagCD*	Forward Reverse	ACACCCGGTGCWWTGAA CAGTGCCACYTTAATCTYC	581 bp
*hicAB*	Forward Reverse	GCAGCAACCATTTGAAACT CAATTCGCCTGGCTAACT	465 bp
*chpBIK*	Forward Reverse	CGCTACTCGCTTGATGAA GCCAGACCAATACGCTTT	397 bp
*mqsRA*	Forward Reverse	AACGCACACCACATACAC AGGATGAGGTTGGGCATT	624 bp
*mazEF*	Forward Reverse	ATGATCCACAGTAGCGTAA TCTTCGTTGCTCCTCTTG	519 bp
*pemIK*	Forward Reverse	ACAGTGTGATCCGAATGC TCAAGTCAGAATAGTGGACAG	416 bp
*parDE*	Forward Reverse	GGCAAGACCATTAAGCAATA GAGTTCGCTCATGTCCTT	350 bp
*relBE*	Forward Reverse	TAGCATTAACCTGCGTATTG TACTTCCGAGCGTTCTCT	492 bp
*ccdAB*	Forward Reverse	AAGCAGCGTATTACAGTGA CCATATCGGTGGTCATCAT	422 bp
*dinJyafQ*	Forward Reverse	GCGAGCCTAATCAATTAACC GCGTGAGTTCCAGTTCTC	372 bp

The primers taken from Fiedoruk et al. (2015).

### Phylogenetic group determination

Bacterial DNA extracted by boiling method was used as a template for PCR. The phylogenetic groups of *E. coli* isolates were determined by amplifying fragments of the chuA (279 bp) and yjaA (211 bp) genes, and the DNA fragment TspE4.C2 (152 bp) by a triplex PCR as described by Clermont et al. (2000). Briefly, groups were assigned based on different combinations of presence and/or absence of the three amplicons as follows: chuA (−) TspE4.C2(−), group A; chuA (−) TspE4.C2(+), group B1; chuA (+) yjaA (−), group D; and chuA (+) yjaA (+), group B2. The primers used are shown in Table 3.

**Table 3:** Primer sequences of genotyping of *E. coli.*

		Direction	Primers 5′–3′	Product size
Phylogenetic grouping^a^	*chuA*	Forward Reverse	ATGATCATCGCGGCGTGCTG AAACGCGCTCGCGCCTAAT	286 bp
	*yjaA*	Forward Reverse	TGTTCGCGATCTTGAAAGCAAACGT ACCTGTGACAAACCGCCCTCA	216 bp
	*TSPE4.C2*	Forward Reverse	GCGGGTGAGACAGAAACGCG TTGTCGTGAGTTGCGAACCCG	152 bp
MLST^b^	*adk*	Forward Reverse	TGCCATTAACCGTTTCAGCC CCGTCAACTTTCGCGTATTT	583 bp
	*fumc*	Forward Reverse	TCACAGGTCGCCAGCGCTT GTACGCAGCGAAAAAGATTC	806 bp
	*gvrb*	Forward Reverse	TCGGCGACACGGATGACGGC ATCAGGCCTTCACGCGCATC	911 bp
	*icd*	Forward Reverse	ATCGAAAGTAAAGTAGTTGTTCCGGCACA GGACGCAGGATCTGTT	878 bp
	*mdh*	Forward Reverse	ATGAAAGTCGCAGTCCTCGGCGCTGCTGGCGG TTAACGAACTCCTGCCCCAGAGAGCGATACTTTCTT	932 bp
	*purA*	Forward Reverse	CGCGCTGATGAAAGAGATGA CATACGGTAAGCCACGCAGA	816 bp
	*recA*	Forward Reverse	CGCATTCGCTTTACCCTGACC TCGTCGAAATCTACGGACCGGA	780 bp

The primers taken from ^a^Fiedoruk et al. (2015). ^b^Tartof et al. (2005). MLST, multilocus sequence typing.

### Multilocus sequence typing

Based on a previously standardized protocol about multilocus sequence typing (MLST) (Tartof et al., 2005), Seven housekeeping genes were analyzed by PCR reaction in 44 rUTI versus 44 UTI strains. The amplification products were sequenced using the Big Dye Terminator v3.1 Cycle Sequencing Kit (Applied Biosystems Inc. Foster City, CA, United States). Sequencing results were submitted to a web-based database^1^ and compared online to obtain allele profiles to obtain the resultant sequence type. The PCR amplification setup and materials were the same as the phylogenomic analysis described above, and the primer sequences used for MLST are listed in Table 3.

### Plasmid construction

The plasmids were constructed using standard molecular biology techniques (Sambrook and Russell, 2001). The *ccdA*, *ccdB*, *ccdAB* gene was specifically amplified from Clinical strains (GN230320) with PCR. pET28a (+) was recovered and purified by enzymatic digestion with XhoI with EcoRI (NEB, Ipswich, MA, United States) for 3 h at 37°C in a water bath chamber followed by inactivation at 65°C for 20 min and recovered by purification with a PCR purification kit. Design of homology arm primers containing enzyme cleavage sites (*ccdAB*-F:5′-GTGGTGGTGGTGGTGCTCGAATGAAGCAGCGTATTACAGTG-3′; *ccdAB*-R:5’-ATGGGTCGCGGATCCGAATTTTATATTCCCCAGAACATCAGGTTAA-3′,*ccdA*-R:5′-ATGGGTCGCGGATCCGAATTTCACCAGTCCCTGTTC-3′; *ccdA*-F same as *ccdAB*-F; *ccdB*-F:5′-GTGGTGGTGGTGGTGCTCGAATGCAGTTTAAGGTTTACACC-3′)*ccdB*-R same as *ccdAB*-R. Constructs pET28a-*ccdAB*, pET28a-*ccdA*, and pET28a-*ccdB* were carried out using homologous recombinase (Tiangen, Beijing, China) at 50°C and distributed for transformation into *E. coli* BL21. Colony PCR and subsequent sequencing were used to validate constructed recombinant plasmids.

### Bacteria growth curves

In this study, we meticulously analyzed the growth curves of four *E. coli* BL21 strains with different plasmids: BL21-pET28a-*ccdAB*, BL21-pET28a-*ccdA*, BL21-pET28a-*ccdB*, and BL21-pET28a. The procedure began with overnight cultures of each strain being diluted in a 1:100 ratio into 10 mL of fresh LB broth. The growth of these cultures was closely monitored until they reached the logarithmic phase, indicated by an optical density at OD_600_ of approximately 0.3. The absorbance was measured using an enzyme-linked immunosorbent assay (ELISA) plate reader (Spectra Max; Molecular Devices). IPTG was added at a final concentration of 1 mmoL/L and growth was carried out at 37°C with shaking at 200 rpm. Cell density was measured by OD_600_ every 1 h for a total of 10 h. The experiment was designed for three independent experiments.

### Biofilm formation assay

Biofilm formation was estimated by crystal violet (CV) assay as previously described (Fu et al., 2018). Single colonies of BL21-pET28a-*ccdAB*, BL21-pET28a-*ccdA*, BL21-pET28a-*ccdB*, and BL21-pET28a were picked and grown overnight in LB broth at 37°C with shaking at 200 rpm. The OD_600_ value was determined at 1.0–1.5 by passaging and inoculating into fresh LB broth at a ratio of 1:100, then 200ul of culture was transferred to a 96-cell polystyrene microtiter plate. After 48 h of incubation in a 37°C incubator, the culture solution was discarded, washed with 200ul of sterile PBS 1–2 times, fixed with 200ul of 99% methanol for 15 min, and then discarded, and naturally air-dried at room temperature. The adherent biofilm was stained with 1% crystal violet solution for 5 min, then the excess dye was rinsed off with running water, dried at room temperature, and dissolved by adding 200 ul of 33% glacial acetic acid, and placed in a 37°C thermostat for 30 min. Finally, biofilm quantification was measured at 590 nm using an enzyme-linked immunosorbent assay (ELISA) plate reader (Spectra Max; Molecular Devices). Absorbance data were obtained from three replicate experiments.

### Persistence assay

Bacterial Persistence culture protocols have been previously described (Liu et al., 2017). In this study, we employed a time-dependent killing assay to evaluate the persistence of the *ccdAB*. To ensure that the remaining TA systems in the two strains (GN23320, GN23616), one containing *ccdAB* and the other not, remained the same, we performed PCR experiments to amplify the most widely studied persistence-related TA systems for screening, and found that the two strains remained consistent in distribution and content. The process began with the selection of fresh single colonies (GN23320, GN23616), which were incubated in 5 mL LB medium at 37°C with a shaking speed of 200 rpm overnight. These overnight cultures were then diluted at a 1:100 ratio into fresh LB medium. The growth was monitored until an optical density (OD_600_) of 0.6 was reached. At this stage, to test the specified bacterial strain’s state, we added the antibiotic imipenem to the culture at a concentration of 20 x MIC (5.0 μg/mL). This point was marked as the 0 h of the assay. Subsequently, serial dilutions were performed and samples were plated every 2 h up to a total duration of 10 h.

### Total RNA isolation, cDNA generation, and quantitative real-time reverse-transcription PCR

To validate the function of *ccdAB*, total RNA was extracted from both the original bacteria and the bacteria being held for validation. The original bacteria containing *ccdAB* and the bacteria that remained after 10 h of treatment with antibiotics were picked and incubated in LB medium at 37°C and 200 rpm overnight. The next day, a 1:100 dilution shaking culture was carried out to reach the logarithmic phase of growth, with OD600 ranging between 0.6 and 0.8. Cells were collected and total RNA was extracted using the TransZol Plus RNA kit (TransZol Up Puls RNA Kit, ER501, TransGen, China). cDNA was generated by reverse transcription reactions using (RT mix with Dase (All in one), UE, China) according to the manufacturer’s instructions. Gene expression differences were normalized using the 2-ΔΔCt method and 16srRNA expression. All qRT-PCR assays were repeated at least three times. Primers used for qRT-PCR are listed in Table 4.

**Table 4:** Primers used for qRT-PCR.

Gene name	Direction	Primers 5′–3′
16sRNA	Forward	CGGTGAATACGTTCCGG
	Reverse	CGGTGAATACGTTCCGG
*ccdAB*	Forward	CGGCTCTTTTGCTGACGAGA
	Reverse	CTCTGTACATCCACAAACAG

### Statistical analysis

We conducted independent samples t-tests to examine variations in the distribution of toxin-antitoxin systems between recurrent and nonrecurrent urinary tract infections, disparities between biofilms, and distinctions in primitive versus persistent mRNAs. Additionally, we employed chi-square tests to assess variations in genes. Plots were generated using GraphPad Prism 8 software (La Jolla, CA, United States), while statistical results were analyzed using SPSS 22 software (Chicago, IL, United States). The significance threshold for all statistical tests was established at a *p*-value of 0.05.

## Results

### Sample collection

Eighty eight strains of urinary tract infections (UTIs) were collected from the First Affiliated Hospital of Ningbo University, of which 44 strains were recurrent UTIs and the remaining were non-recurrent UTIs. The rUTI strains originated from urology (48%), nephrology (19%), endocrinology (14%), infectious diseases (6%), emergency medicine (6%), geriatrics (2%), gynecology (2%), and neurology (2%). All endocrinology patients had a history of diabetes of varying degrees.

**Figure 1:**
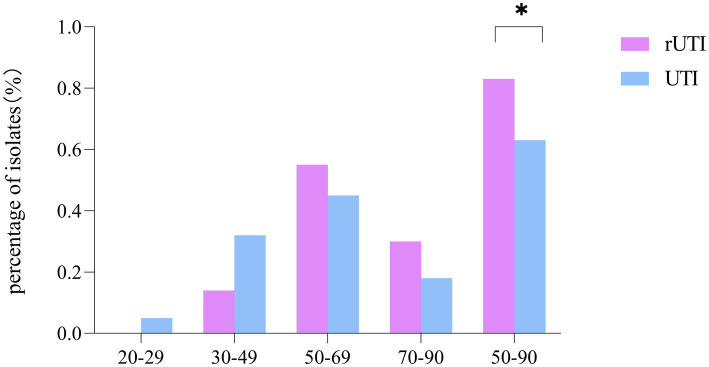
Comparison of age distribution of recurrent and non-recurrent urinary tract infections. SPSS 26.0. *p* < 0.05 (marked *) was defined as a significant difference.

Regarding the age distribution of rUTI patients, it was observed that the age groups of 20–29 years had no cases (0%), 30–49 years had 6 cases (14%), 50–69 years had 24 cases (55%), and 70–90 years had 14 cases (30%). In contrast, the age distribution for non-recurrent UTI patients was as follows: 20–29 years with 2 cases (5%), 30–49 years with 14 cases (32%), 50–69 years with 20 cases (45%), and 70–90 years with 8 cases (18%). A significant observation was that menopausal women, aged between 50 and 90 years, constituted the majority of the rUTI cases, accounting for 83% as compared to 64% in non-recurrent UTIs, a statistically significant difference (*p* < 0.05) as illustrated in Figure 1.

### Toxin-antitoxin system

**Figure 2:**
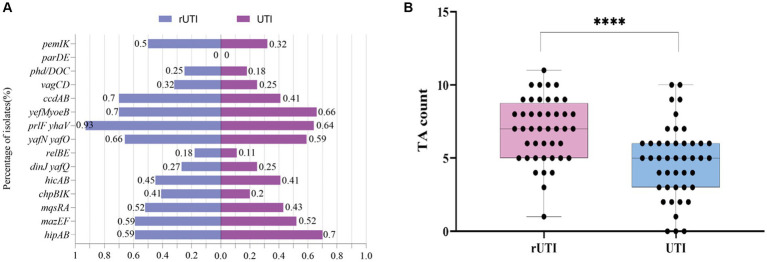
**(A)** Distribution of 15 pairs of toxin-antitoxin genes in rUTI versus UTI. **(B)** TA counts for rUTI vs. UTI, with each dot representing the amount of TA contained in each strain. They were calculated using independent samples *t*-test, SPSS 26.0. *p* < 0.0001 (labeled ****) was defined as extremely significant.

Of the 15 TA loci tested, the TA loci *parDE* was not detected either in rUTI or UTI (Figure 2A), where *ccdAB*, *chpBIK*, *mqsRA*, and *prlfyhav* were statistically different in distribution between rUTI and UTI, and the others were not statistically significant (Table 5). Of the 15 pairs of genomes tested, the number of toxin-antitoxin systems present in rUTI ranged from a minimum of 1 to a maximum of 11, with a median of 9, whereas the number of TA counts detected in UTI ranged from 0 to 10, with a median of 4, analyzed using an independent samples t-test at *p* < 0.001 (Figure 2B).

**Table 5:** Distribution of 15 pairs of toxin-antitoxin genes in rUTI versus UTI.

TA	rUTI (*n* = 44)	UTI (*n* = 44)	*p*-values
*ccdAB*	31	18	0.005
*hipAB*	26	31	0.265
*mazEF*	26	23	0.50
*chpBIK*	18	9	0.037
*hicAB*	20	18	0.667
*dinJ yafQ*	12	11	0.323
*relBE*	8	5	0.367
*yafN yafO*	29	26	0.509
*prlF yhaV*	41	28	0.001
*yefMyoeB*	28	16	0.509
*vagCD*	14	11	0.478
*phd/doc*	11	8	0.437
*parDE*	0	0	-
*pemIK*	22	14	0.083
*mqsRA*	23	19	0.039

The distribution of 15 toxin-antitoxin systems between 44 rUTIs and 44 UTIs was statistically analyzed using the chi-square test, SPSS 26.0 software.

### Phylogenetic group determination

**Figure 3:**
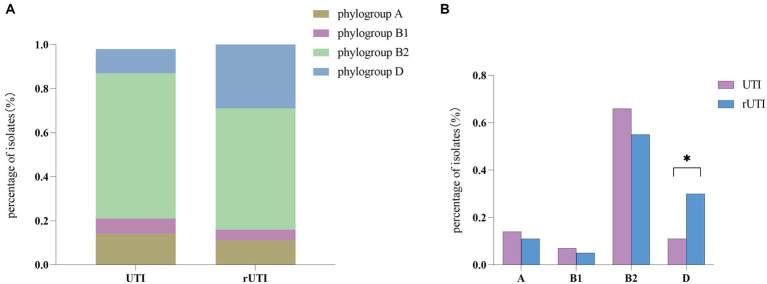
**(A)** Comparison between phylogenetic clusters of recurrent and nonrecurrent urinary tract infections. Abbreviations: rUTI, Recurrent urinary tract infections; UTI, urinary tract infections. **(B)** Differential comparison between rUTI and UTI phylogenetic groups. B2, phylogenetic group B2; B1, phylogenetic group B1; A, phylogenetic group A; D, phylogenetic group D Calculated by ANOVA, one-way ANOVA, SPSS 26.0. *p* < 0.05 (marked *) was defined as the significant difference.

**Figure 4:**
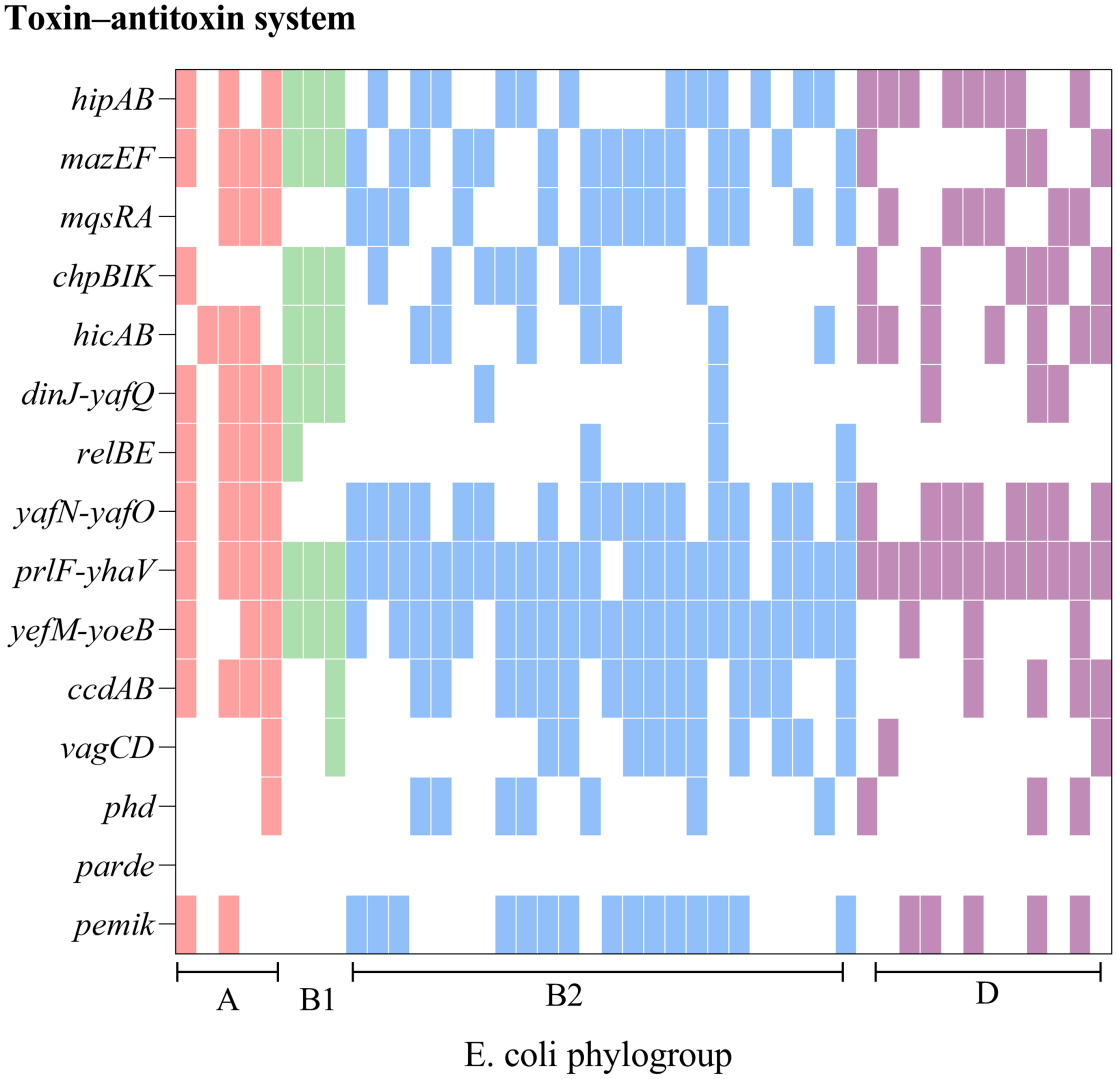
Comparison of chromosomes and toxin-antitoxin locus between rUTI strains. The figure shows the PCR-mediated detection of 15 chromosomal toxin-antitoxin systems in 44 *E. coli* distributed across four phylogroups (A, B1, B2, D) and shows that toxin-antitoxin systems are seldom conserved from one strain to another, even within the same phylogroup.

From Figure 3 it is seen that rUTI and UTI developmental groups were mainly B2, *E. coli* causing rUTI was more common in phylogenetic group B2, *n* = 24 (55%) followed by D, *n* = 13 (30%) A, *n* = 5 (11%) B1, *n* = 2 (5%). Similarly in the UTI group B2 was most common, *n* = 29 (66%) followed by A, *n* = 6 (14%) D, *n* = 5 (11%) B1, *n* = 3 (7%). And it can be seen that rUTI *E. coli* was significantly more prevalent in group D (30% vs. 11%), *p* < 0.05 (Figure 3B). Groups A and B1 showed no significant association with the course of infection. Some toxin-antitoxin systems show quasi-universal conservation across one phylogroup or several (for example, *prlF-yhaV*), indicating a vertical transmission of these systems within phylogroups (Figure 4).

### MLST typing result

**Figure 5:**
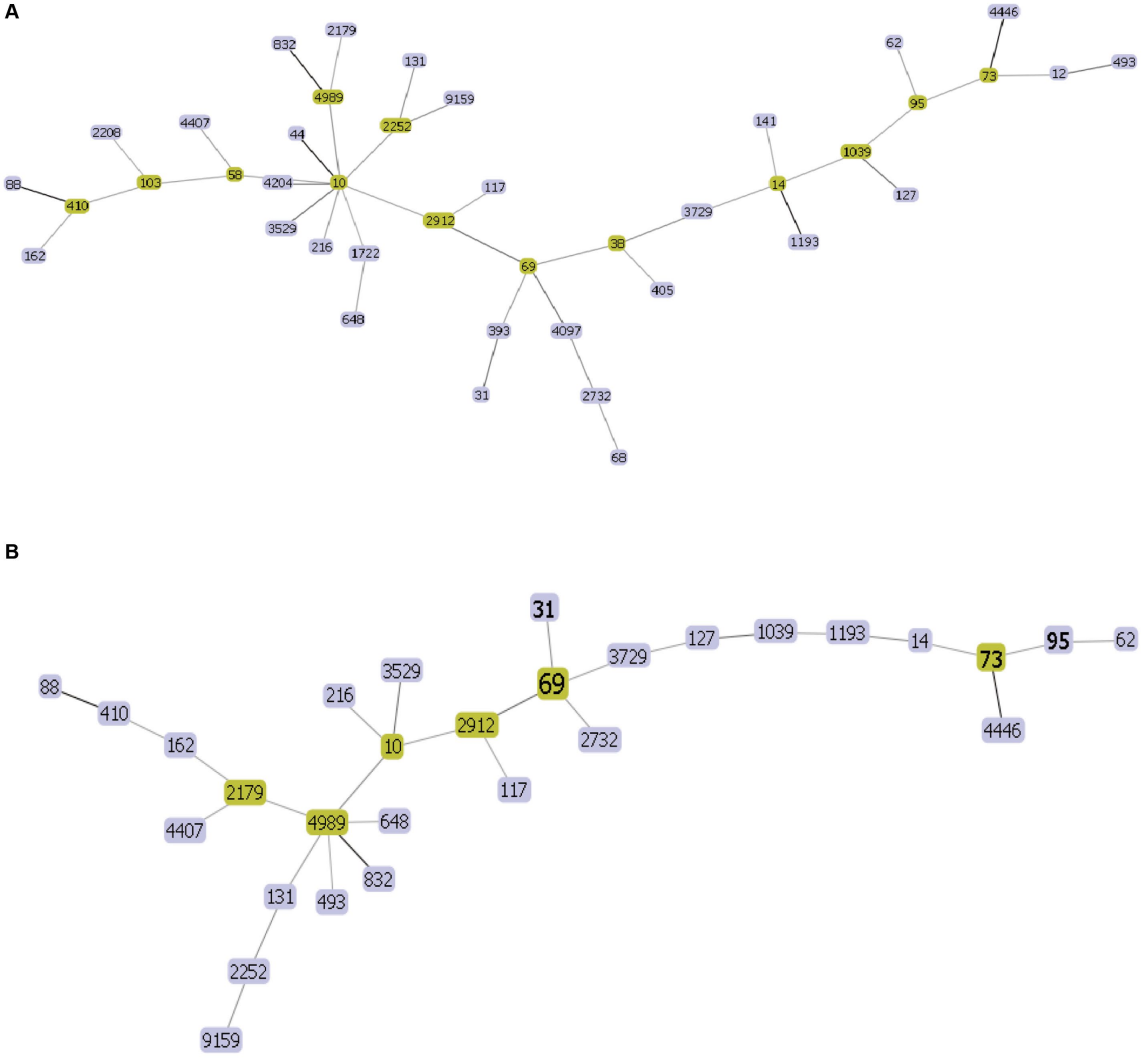
UPEC MLST minimum spanning tree each circle represents a sequence type (ST), and the line between each circle indicates the relationship between different STs. **(A)** Minimum spanning tree for rUTI MLST **(B)** minimum spanning tree for UTI MLST. Note: Yellow color indicates clonal complexes, purple color is their branches.

The 44 rUTI strains were categorized into 21 sequence types, of which the first ST types were ST131, accounting for 18.2% (8/44), ST1193, accounting for 18.2% (8/44), followed by ST69, accounting for 11.4% (5/44), and ST38, ST14, ST1722, ST648, each accounting for 4.5% (2/44), and lastly, ST393, ST44, ST2208, ST405, ST4097, ST58, ST12, ST393, ST44, ST2208, ST405, ST4097, ST103, ST68, ST58, ST12.ST4204, ST4989, ST9159, ST832, and ST141 were one strain each. A total of 27 sequence types were categorized among the 44 UTIs, of which the first one was ST69 with 11.4% (5/44) followed by ST73 and ST1193 with 9.1% (4/44), ST95 with 6.8% (3/44), ST31, ST10 and ST2252 with 4.5% (2/44) and finally ST103, ST62, ST2912, ST4452, ST4204, ST4989, ST9159, ST832, and ST141 with one strain each. ST2912, ST4446, ST117, ST127, ST1039, ST2732, ST4407, ST410, ST162, ST648, ST131, ST493, ST2179, ST14, ST3729, ST3529 each accounted for one plant. The minimum spanning tree is shown in Figure 5.

### Successful construction of recombinant plasmids

**Figure 6:**
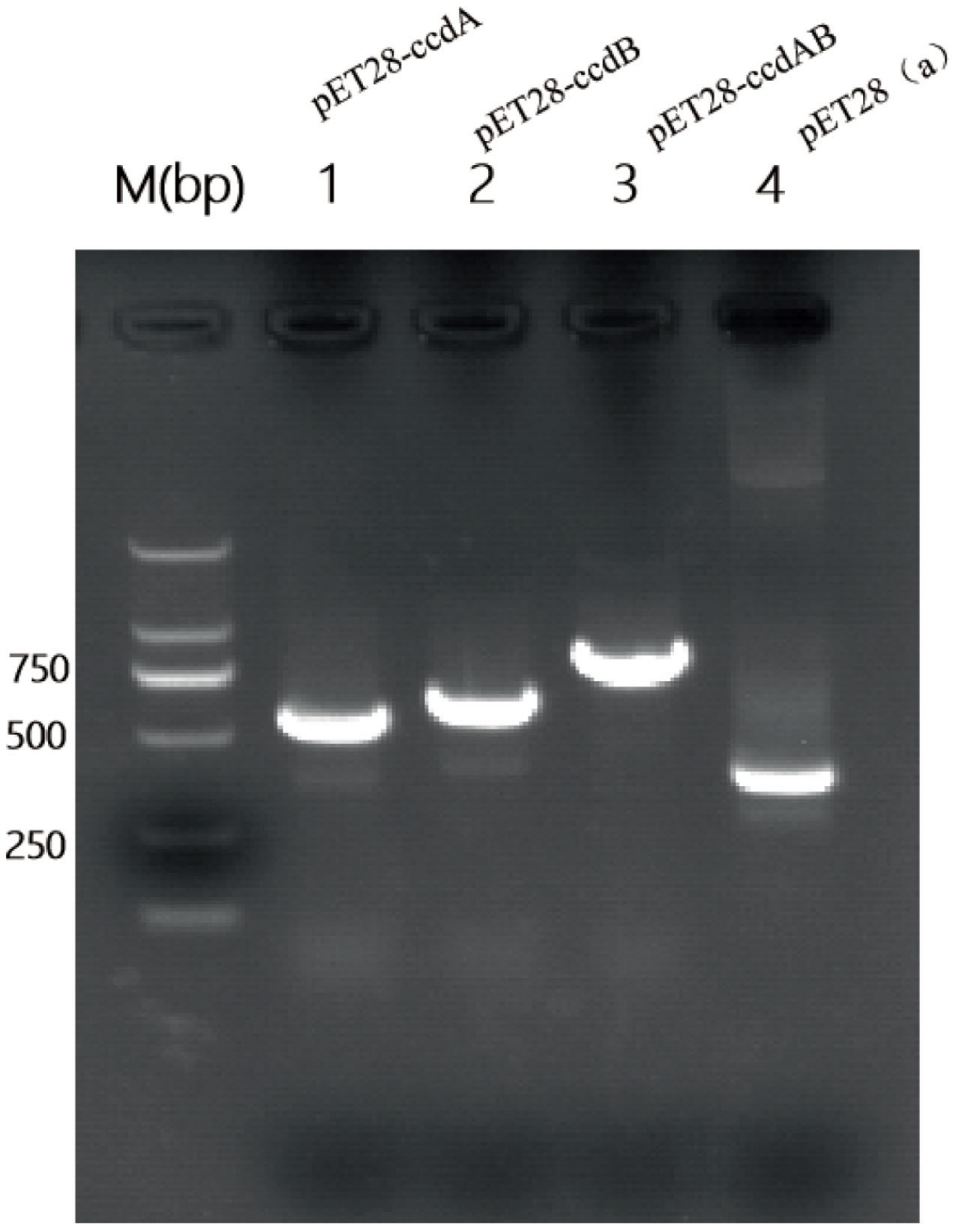
Construction of PCR-amplified mcs-F/R validated recombinant plasmid PCR amplification of mcs-F: 5′-CCAACTCAGCTTCCTTTCGG-3′, mcs-R: 5′-TCGGTGATGTCGGCGATATA-3′ was performed using primers to identify whether the recombinant plasmids were constructed successfully. Where the M value is a 2000 bp marker and lane 4 is a negative control with a size of 339 bp. In the figure, lane 1 is BL21-pET28a-ccdA size 555 bp, lane 2 is BL21-Pet28a-ccdB size 642 bp, and lane 3 is BL21-pET28a-ccdAB size 865 bp.

To investigate the role of *ccdAB*, BL21-pET28a-*ccdAB*, BL21-pET28a-*ccdA*, and BL21-pET28a-*ccdB* were constructed using homologous recombination, and the correct recombinants were confirmed by PCR and sanger sequencing. Primers with homology arm (25 bp) were designed and BL21-pET28a-*ccdAB*, BL21-pET28a-*ccdA*, and BL21-pET28a-*ccdB* were successfully constructed according to the instructions of homologous recombinase, the sizes of which were 865, 555, and 642 bp in that order (Figure 6).

### Expression of the inducible toxin *ccdB* affects bacterial growth

**Figure 7:**
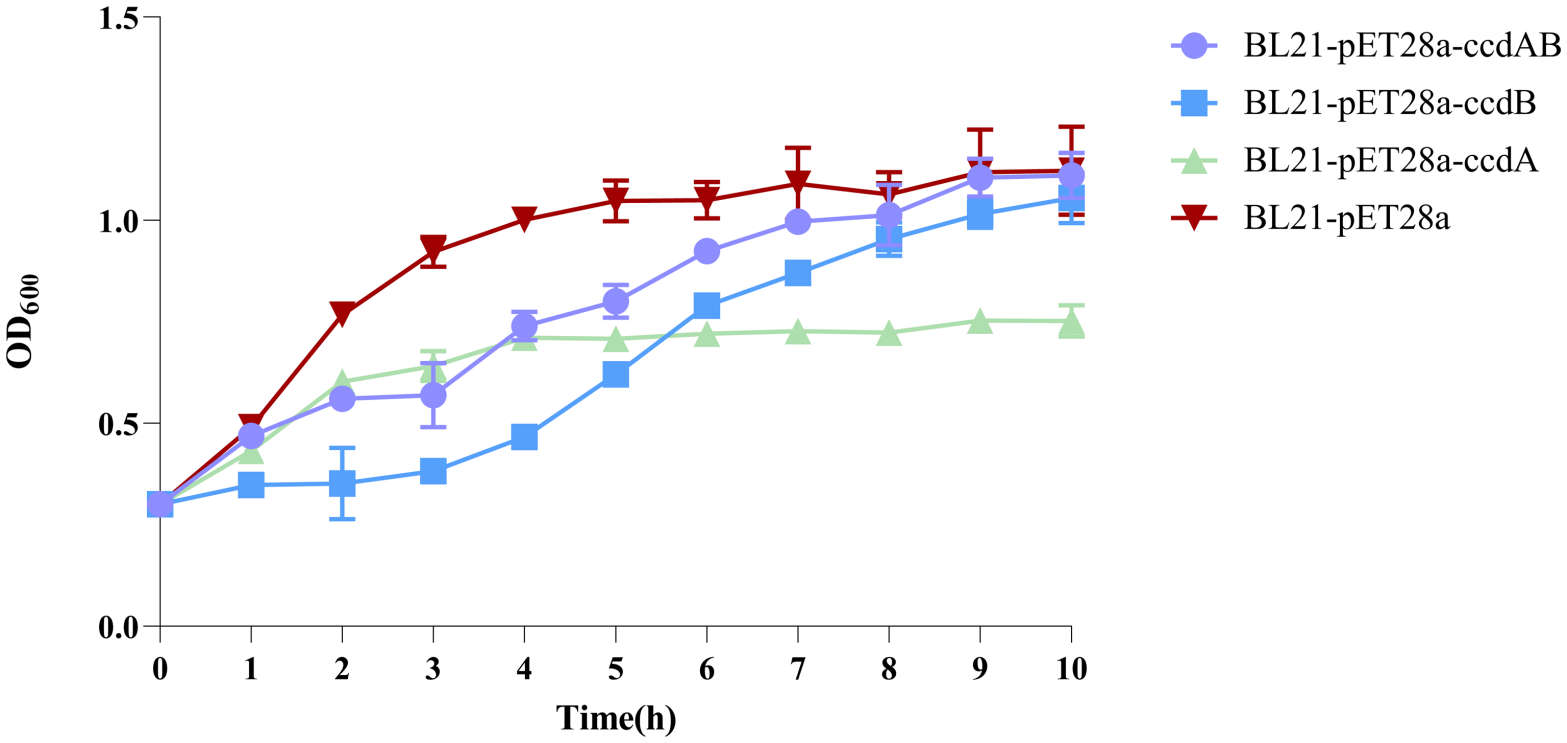
Toxin *ccdB* inhibits bacterial growth. The growth rates of BL21-pET28a-CcdAB, BL21-pET28a-CcdA, BL21-pET28a-CcdB, and BL21-pET28a were similar by measuring the optical density (OD_600_) per hour for 10 h. Data are shown as standard deviation ± mean.

By inducing the expression of the toxin protein *ccdB*, it was found that the OD value of the toxin *ccdB* was significantly lower than that of BL21-pET28a-*ccdAB* with BL21-pET28a from about 1 h of growth, indicating a certain inhibitory effect on the growth of bacteria. When the toxin and the antitoxin were co-induced to be expressed, the antitoxin *ccdA* neutralized the toxic effect of the corresponding toxin to a certain extent, which could bring the bacteria back to the normal growth state, whereas when the antitoxin *ccdA* was expressed alone, the bacteria did not continue to grow from the 4th hour onwards, and were in a stable growth state (Figure 7).

### *ccdAB and ccdB* increase biofilm formation

**Figure 8:**
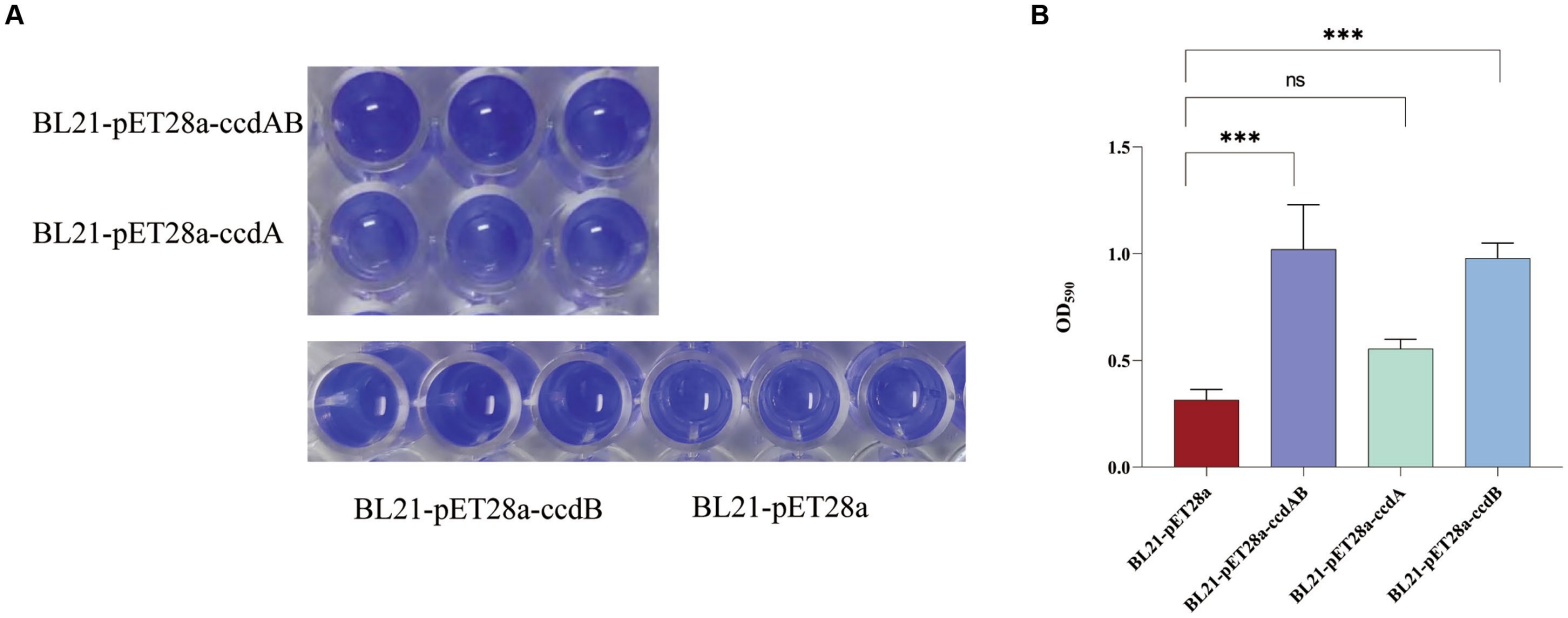
**(A)** Image of biofilm formed by BL21-pET28a-ccdAB, BL21-pET28a-CcdA, BL21-pET28a-CcdB, and BL21-pET28a.biofilm was stained with 1%CV, washed with sterile water and then dissolved with 33% glacial acetic acid. **(B)** the extracted color was measured at OD590 to show biofilm production. Asterisks (***) represent statistically significant differences in biofilm formation between BL21-pET28a and BL21-pET28a-CcdAB/BL21-pET28a-CcdB (*p* < 0.001, *t*-test). The biofilm formation of BL21-pET28a and BL21-pET28a-CcdA is not statistically significant.

In this study, we conducted a CV staining assay to investigate its impact on biofilm formation in a strain of rUTI caused by UPEC. As shown in Figure 8A, BL21-pET28a-*ccdAB* and BL21-pET28a-*ccdB* solids indicate an increase in the associated biofilm formation, and BL21-pET28a-*ccdA* has an insignificant increase in the formation of biofilm, which is an about two-fold increase compared to the control BL21-pET28a (Figure 8B), suggesting that the genes *ccdAB* and *ccdB* can promote bacterial biofilm formation. *ccdAB* has been reported to regulate *E. coli* Nissle 1917 (EcN) biofilm formation (Xu et al., 2020), which aligns with our research results. In this study, *ccdAB* was derived from clinical strains and combined with urinary tract infections to provide a new target for clinical therapy.

### *ccdAB* affect persistence

**Figure 9:**
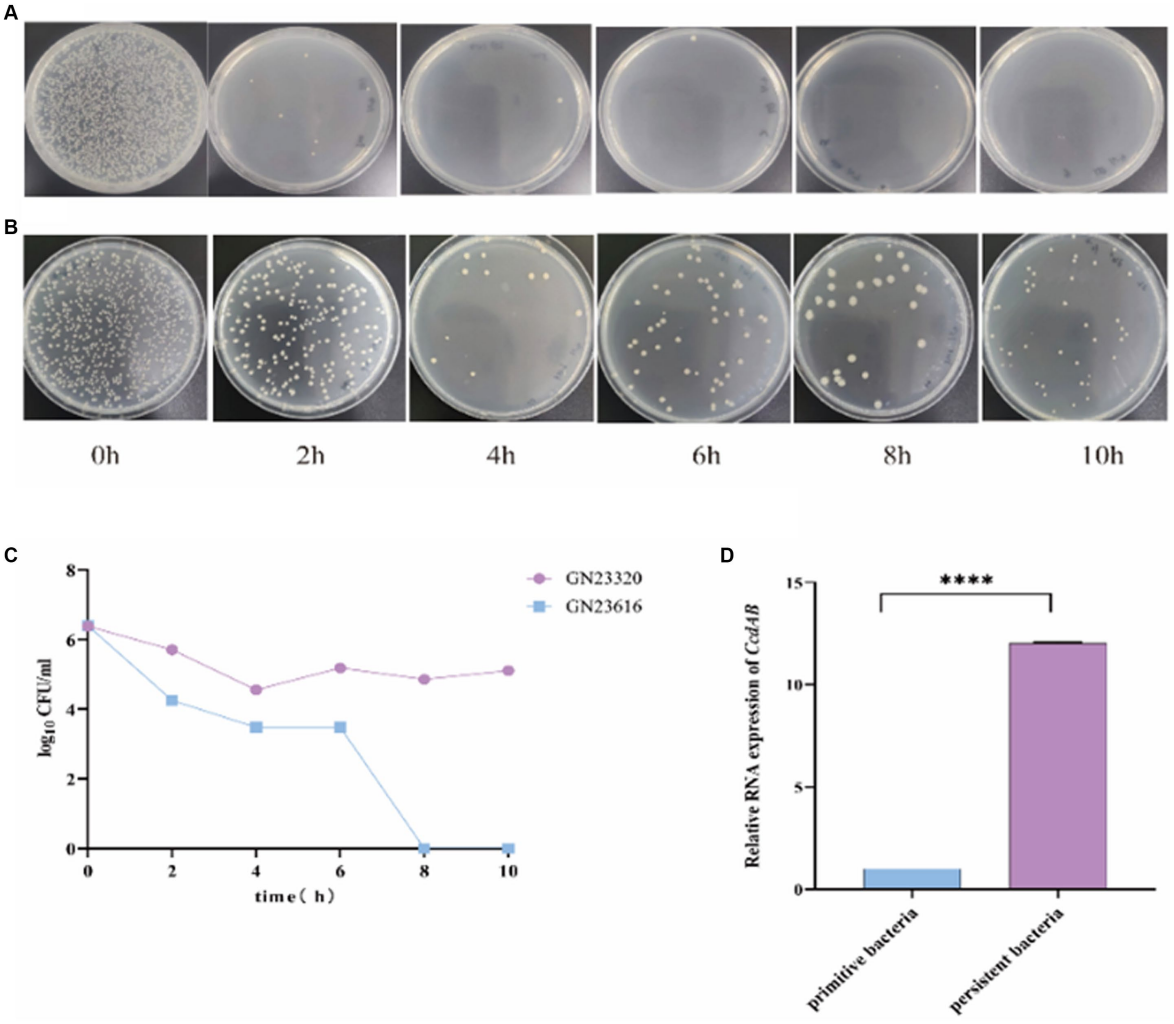
Detection of rUTI persistent Bacteria. The images depict the persistent bacteria assay conducted on strains GN23616 **(A)** and GN23320 **(B)** using high-dose imipenem. GN23320 contains *CcdAB* while GN23616 does not. **(C)** The number of bacterial colonies was observed to vary with time at a dilution of 10^−3^. **(D)** RNA was extracted from both the primitive and the persister bacteria, and the quantification of changes in the multiplicity of expression of the *CcdAB* genes was performed using qRT-PCR (*p* < 0.0001 was shown in ****, *n* = 3).

To investigate the role of *ccdAB* in rUTI, we conducted persister cell tests. Persistence cell analyses were performed on clinical strains containing *ccdAB* and those without it, using a high dose of imipenem. The time-dependent killing assay, shown in Figure 9A, revealed that in *ccdAB*-free *E. coli*, the number of colonies began to decrease from the fourth hour onwards. Single colonies started to appear and then ceased growing. In contrast, the *ccdAB*-containing strain showed the bimodal killing curve, indicating the emergence of persistent bacteria (Figure 9C). To confirm the function of *ccdAB*, we extracted the original colonies from *ccdAB*-containing strains and those that remained after 10 h of growth for qRT-PCR verification (Figure 9D). The statistical analysis yielded a *p*-value of less than 0.0001.

## Discussion

In this study, we investigated the variations in TA distribution, TA number, phylogenetic distribution, and MLST typing among rUTIs caused by UPEC. We found that bacterial growth was inhibited when *ccdB* expression was induced in *E. coli* BL21 (DE3). Furthermore, the expression of *ccdAB* and *ccdB* led to an increase in biofilm formation in *E. coli* BL21 (DE3), confirming that *ccdAB* induced the formation of persistent bacteria in urinary tract infections.

A better understanding of the characteristics that predispose pathogens to rUTI will help develop new therapeutic strategies. Focusing on female inpatients, our research aligns with the finding that rUTIs are among the most prevalent infections in women, affecting up to half of them during their lifetime, with a significant recurrence rate within 6–12 months (Foxman, 2002; Fisher et al., 2017). In this study we find the majority of women with rUTI were menopausal women with an age distribution between 50 and 90 years (83%) (Figure 1). It is clear that menopause is a major risk factor for rUTI in women, which correlates with postmenopausal estrogen levels, making the vaginal environment more susceptible to infection by urinary tract pathogens (Portman and Gass, 2014). Undoubtedly, urologic patients account for the vast majority of rUTI. It is worth noting that about 14% of the rUTI patients in this study had a history of diabetes to varying degrees. Previous research has reported that diabetic patients are more susceptible to urinary tract infections due to the higher level of sugar in their urine, which provides an additional source of nutrients for bacteria to grow and multiply. This aligns with existing literature highlighting the increased UTI risk in diabetic patients, attributed to elevated urinary sugar levels and compromised immune function due to high blood sugar (Bersoff-Matcha et al., 2019). Therefore, it is crucial to focus on this particular group of individuals.

From this experiment, it can be concluded that there is a significant difference between toxin antitoxin systems in recurrent and nonrecurrent urinary tract infections. There was a statistically significant difference between the two groups in the distribution of *ccdAB*, *chpBIK*, *mqsRA*, and *prlf yhav* in the TA system (Table 5). Our interesting findings may be explained by the fact that *E. coli* strains belonging to rUTI contain more TA than those belonging to UTI. The number of toxin antitoxins was significantly higher in rUTI strains than in UTI strains (*p* < 0.001).

Our findings suggest a potential link between type II TA and *E. coli* phylogeny. The phylogenetic distribution among the studied strains favored phylogroups B2 and D, which is consistent with the findings of other authors (Massot et al., 2016; Micenková et al., 2016). Our results indicate that genes of the type II TA system were predominant (55%) in phylogenetic group B2, while there was a statistical difference in phylogenetic group D between rUTI and UTI. Group B2 represents the ancestral branch, whereas groups A and B1 are the youngest sister groups in the *E. coli* phylogeny (Lecointre et al., 1998). On the other hand, group D is polyphyletic and consists of an ancestral subgroup and another sister branch of the A/B1 branch. In terms of *E. coli* lifestyle or host preference, group B2 is more likely to be an “expert” in host adaptation (White et al., 2011). Therefore, the higher number of strains in group B2 may reflect their adaptation to the urinary tract environment, leading to persistent rUTI. In addition, Others appear sporadically across the phylogeny with no clear patterns (for example, *relBE* or *phd*), indicating multiple acquisition events through horizontal gene transfer (Ramisetty and Santhosh, 2016; Figure 4). The variation in the phylogenetic distribution of UPEC strains may be influenced by host specificities such as geographic environment, climate, and diet type, as well as other predisposing factors like anatomical alterations, metabolic disorders, immune status, and hygienic practices.

According to recent surveys, the two most prevalent sequence types of *E. coli* urine isolates in which the presence of the ESBL gene was detected by PCR were ST131 and ST1193 (Ejrnæs et al., 2011). Our results are consistent with this finding. The ST131 clone has emerged as the most important and potent multidrug resistance (MDR) clone globally and is the leading cause of community-acquired urinary and bacteremia infections. The ST1193 clone closely follows due to its widespread use of fluoroquinolones (FQs) and third-generation cephalosporins (Fukushima et al., 2021). Birgy et al. found that between 2014 and 2017, out of 218 ESBL-producing *E. coli* infections resulting in febrile urinary tract infections in children, ST1193 was one of the most prevalent clones in the final study period.

Toxins with toxic effects are expressed to inhibit bacterial growth, while antitoxins can neutralize their toxic effects and restore growth. In this study, it was found that bacterial growth was inhibited when the toxin *ccdB* was induced to be expressed, whereas *ccdAB* co-expression inhibited the corresponding toxin to a certain extent, but still did not reach normal growth status. The bacterial growth was arrested when the antitoxin *ccdA* was induced alone, probably because the expression of the antitoxin in this TA system disrupted the equilibrium of some of the TA systems within the bacteria, resulting in a disruption of the bacterial metabolism and thus growth arrest. The interaction between *ccdB* and *ccdA* was found to be dependent on the concentration ratio between the two: when the ratio between *ccdA* and *ccdB* is greater than or equal to 1, the *ccdA* dimer forms a complex with the *ccdB* dimer (*ccdA*2-*ccdB*2), and *ccdA* has an inhibitory effect on *ccdB*: whereas when the ratio between *ccdA* and *ccdB* is less than 1, a complex consisting of one *ccdA* dimer and two *ccdB* dimers form a complex (*ccdA*2-*ccdB*4), and *ccdA* has no inhibitory effect on *ccdB*; we call this phenomenon conditional synergism (Afif et al., 2001). By this mechanism, TA transcription can be dynamically adjusted according to intracellular toxin concentrations and may play a role in maintaining and/or resuscitating persistent cells, leading to persistent urinary tract infections.

Biofilm formation is a good survival strategy for bacterial adaptation in adversity. Clinically, biofilms play an important role in persistent and chronic infections by reducing immune responses and antimicrobial efficacy (Wei and Ma, 2013). The search for new drugs targeting biofilms may lead to new strategies to control infections and provide solutions to biofilm-related problems. Different studies have shown that recurrent urinary tract infections are associated with biofilm formation (Falk et al., 2022; Nasrollahian et al., 2022). Therefore, we investigated the biofilm-forming ability of *ccdAB* in rUTI induced by *E. coli*. As the first TA to be characterized and well-studied, the plasmid-encoded *ccdAB* is primarily involved in plasmid maintenance through a process known as post-segregation killing. The chromosomal *ccd* operon of *E. coli* O157 is involved in drug tolerance and provides cell death protection during exposure to multiple antibiotic stresses. Our findings that the *ccdAB* module promotes stronger biofilm formation may explain why certain *E. cc* strains are particularly difficult to clear in the urinary tract environment. This study suggests that further work is needed to fully understand the mechanism by which *ccdAB* promotes biofilm formation in rUTI.

In this study, we discovered that *E. coli* strains containing the *ccdAB* gene complex contributed significantly to bacterial persistence in rUTIs. Under stress conditions, the antitoxin *ccdA* is degraded by the Lon protease. This releases the toxin *ccdB*, which then interferes with cellular processes by poisoning the DNA-polymerase complex and causing double-strand DNA breaks. Our findings suggest that targeting the *ccdAB* system may be a novel approach for the treatment of acute urinary tract infections, especially when bacteria form biofilms that are more likely to form persistent bacteria. As noted by Wang et al. (2015), biofilm formation is a crucial strategy for bacterial persistence in the urinary tract. Our study opens avenues for exploring how other toxin-antitoxin systems might contribute to bacterial persistence in rUTIs. Persistent bacteria are a key factor in recurrent urinary tract infections, and while most bacteria are killed after antibiotic treatment, persistent bacteria can survive, and when treatment ends or environmental conditions change, these persistent bacteria can reactivate, leading to recurrent infections (Defraine et al., 2018). In some cases, the persistent bacteria may lead to chronicity of urinary tract infections. These bacteria can survive in the host for a longer period, leads to a long-team, low-intensity infection.

As rUTI is characterized by recurrent attacks, difficult to cure and not easy to be cured, it leads to mental tension, anxiety, and even depression, bringing heavy physical and mental pain to patients and their families. Long-term recurrent infections or prolonged treatment can eventually lead to chronic renal failure. However, many pathogenic bacteria have evolved a complex set of survival mechanisms capable of evading the immune response and developing persistent infections without being cleared by the body’s immune response and antimicrobial drugs (Monack, 2013).

In recent years, the type II toxin-antitoxin system has been the focus of attention in the study of mechanisms of holdout formation. In the same seminal microfluidic study, Balaban and his colleagues (Balaban et al., 2004) also used another *E. coli* hip mutant: the *hipQ* strain. Brennan and coworkers (Schumacher et al., 2015) screened a library of 477 *E. coli* isolates, including commensals and UTI patients, for *hipA* mutations, and showed that, in addition to the importance of the hipA7 mutation in persistent formation *in vitro* in *E. coli*, highly persistent *hipA* mutations, including hipA7, were selected for over time in patients with rUTI. Highly persistent mutants selected *in vitro* through cycles of antibiotic exposure have been repeatedly identified in the TA module, and the *hipA* mutation has been shown to accumulate over time in clinical isolates of *E. coli* from patients with recurrent UTI. Interestingly, several other studies have shown a direct link between the persistence phenomenon and the TA motif. Two studies have shown that persistent cells contain elevated levels of TA mRNA (Shah et al., 2006). Two other studies have shown that deletion of the TA motif reduces persistence in biofilms and during the induction of the SOS response (Dörr et al., 2010).

It is worth noting that a limited number of *E. coli* isolates were selectively obtained from a single tertiary care hospital. Therefore, these findings are not generalizable to all rUTI strains. Therefore, we hope to utilize a multicenter approach for extensive molecular epidemiology studies in future efforts.

Our study further reveals the specific role of the *ccdAB* module in rUTI. Due to the role of the *ccdAB* module in biofilm formation and bacterial persistence, it may be a key factor in the survival of *E. coli* in urinary tract infections and in causing recurrence. This adaptation to the environment may be one of the reasons why *E. coli* has become a major pathogen of rUTI. Future studies need to further explore how the *ccdAB* module mutates in different *E. coli* strains and how these mutations affect bacterial pathogenicity and response to treatment. In addition, the development of specific inhibitors against the *ccdAB* module will be an important research direction, which may provide a new strategy for the treatment of rUTIs.

## Conclusion remarks

In conclusion, we identify the *ccdAB* gene complex as a significant contributor to bacterial persistence in rUTIs. The study highlights the potential of targeting the *ccdAB* system as a novel approach to rUTIs, especially those involving biofilm-forming bacteria. This research not only adds to our understanding of bacterial persistence in rUTIs but also opens avenues for future exploration into how other toxin-antitoxin systems might contribute to this persistence. As rUTIs are characterized by recurrent episodes and resistance to treatment, leading to significant physical and mental distress, insights from this study could be vital in developing new strategies to combat these infections.

Currently, TA systems have been developed and utilized in biotechnology and biomedicine, and a series of valuable advances have been made in molecular biology and microbiology.

## Data availability statement

The original contributions presented in the study are included in the article/supplementary material, further inquiries can be directed to the corresponding authors.

## Ethics statement

The studies involving humans were approved by Ethics Committee of the First Affiliated Hospital of Ningbo University (No. 2023 Study No. 145RS). The studies were conducted in accordance with the local legislation and institutional requirements. Written informed consent for participation was not required from the participants or the participants’ legal guardians/next of kin because what we have taken are leftover specimens left over from the clinical microbiology unit and are not designed for direct study in humans.

## Author contributions

HZ: Writing – review & editing, Writing – original draft, Methodology, Investigation, Formal analysis, Data curation, Conceptualization. ST: Writing – review & editing. HC: Writing – review & editing. YF: Writing – review & editing. YX: Writing – review & editing. LC: Writing – review & editing. FM: Writing – review & editing, Supervision, Project administration. WL: Writing – review & editing, Supervision, Methodology, Funding acquisition.

## Footnotes

^1^ http://mlst.ucc.ie/mlst/dbs/Ecoli

## References

[ref1] Aakre C. D. Phung T. N. Huang D. Laub M. T. (2013). A bacterial toxin inhibits DNA replication elongation through a direct interaction with the β sliding clamp. Mol. Cell 52, 617–628. doi: 10.1016/j.molcel.2013.10.014, PMID: 24239291 PMC3918436

[ref2] Afif H. Allali N. Couturier M. Van Melderen L. (2001). The ratio between CcdA and CcdB modulates the transcriptional repression of the ccd poison-antidote system. Mol. Microbiol. 41, 73–82. doi: 10.1046/j.1365-2958.2001.02492.x, PMID: 11454201

[ref3] Al-Badr A. Al-Shaikh G. (2013). Recurrent urinary tract infections Management in Women: a review. Sultan Qaboos Univ. Med. J. 13, 359–367. doi: 10.12816/0003256, PMID: 23984019 PMC3749018

[ref4] Balaban N. Q. Merrin J. Chait R. Kowalik L. Leibler S. (2004). Bacterial persistence as a phenotypic switch. Science 305, 1622–1625. doi: 10.1126/science.1099390 15308767

[ref5] Bersoff-Matcha S. J. Chamberlain C. Cao C. Kortepeter C. Chong W. H. (2019). Fournier gangrene associated with sodium-glucose Cotransporter-2 inhibitors: a review of spontaneous Postmarketing cases. Ann. Intern. Med. 170, 764–769. doi: 10.7326/m19-0085, PMID: 31060053

[ref6] Choi J. S. Kim W. Suk S. Park H. Bak G. Yoon J.. (2018). The small RNA, Sds R, acts as a novel type of toxin in *Escherichia coli*. RNA Biol. 15, 1319–1335. doi: 10.1080/15476286.2018.1532252, PMID: 30293519 PMC6284582

[ref7] Clermont O. Bonacorsi S. Bingen E. (2000). Rapid and simple determination of the *Escherichia coli* phylogenetic group. Appl. Environ. Microbiol. 66, 4555–4558. doi: 10.1128/aem.66.10.4555-4558.2000, PMID: 11010916 PMC92342

[ref8] Dai W. Zhang Y. Zhang J. Xue C. Yan J. Li X.. (2021). Analysis of antibiotic-induced drug resistance of Salmonella enteritidis and its biofilm formation mechanism. Bioengineered 12, 10254–10263. doi: 10.1080/21655979.2021.1988251, PMID: 34637696 PMC8809914

[ref9001] Dallenne C. Da Costa A. Decré D. Favier C. Arlet G. (2010). Development of a set of multiplex PCR assays for the detection of genes encoding important beta-lactamases in Enterobacteriaceae. J. Antimicrob. Chemother. 65, 490–495. doi: 10.1093/jac/dkp498 20071363

[ref9] Defraine V. Fauvart M. Michiels J. (2018). Fighting bacterial persistence: current and emerging anti-persister strategies and therapeutics. Drug Resist. Updat. 38, 12–26. doi: 10.1016/j.drup.2018.03.002, PMID: 29857815

[ref10] Dörr T. Vulić M. Lewis K. (2010). Ciprofloxacin causes persister formation by inducing the TisB toxin in *Escherichia coli*. PLoS Biol. 8:e1000317. doi: 10.1371/journal.pbio.1000317, PMID: 20186264 PMC2826370

[ref11] Ejrnæs K. Stegger M. Reisner A. Ferry S. Monsen T. Holm S. E.. (2011). Characteristics of *Escherichia coli* causing persistence or relapse of urinary tract infections: phylogenetic groups, virulence factors and biofilm formation. Virulence 2, 528–537. doi: 10.4161/viru.2.6.18189, PMID: 22030858

[ref12] Falk K. N. Satola S. W. Chassagne F. Northington G. M. Quave C. L. (2022). Biofilm production by Uropathogens in postmenopausal women with recurrent and isolated urinary tract infection. Female Pelvic Med. Reconstr. Surg. 28, e127–e132. doi: 10.1097/spv.0000000000001124 34768258

[ref13] Fiedoruk K. Daniluk T. Swiecicka I. Sciepuk M. Leszczynska K. (2015). Type II toxin-antitoxin systems are unevenly distributed among *Escherichia coli* phylogroups. Microbiology (Reading) 161, 158–167. doi: 10.1099/mic.0.082883-0 25378561

[ref14] Fisher R. A. Gollan B. Helaine S. (2017). Persistent bacterial infections and persister cells. Nat. Rev. Microbiol. 15, 453–464. doi: 10.1038/nrmicro.2017.42 28529326

[ref15] Foxman B. (2002). Epidemiology of urinary tract infections: incidence, morbidity, and economic costs. Am. J. Med. 113, 5–13. doi: 10.1016/s0002-9343(02)01054-9 12113866

[ref16] Fu L. Huang M. Zhang X. Yang X. Liu Y. Zhang L.. (2018). Frequency of virulence factors in high biofilm formation Bla (KPC-2) producing *Klebsiella pneumoniae* strains from hospitals. Microb. Pathog. 116, 168–172. doi: 10.1016/j.micpath.2018.01.030, PMID: 29360567

[ref17] Fukushima Y. Sato T. Tsukamoto N. Nakajima C. Suzuki Y. Takahashi S.. (2021). Clonal/subclonal changes and accumulation of CTX-M-type β-lactamase genes in fluoroquinolone-resistant *Escherichia coli* ST131 and ST1193 strains isolated during the past 12 years, Japan. J Glob Antimicrob Resist 27, 150–155. doi: 10.1016/j.jgar.2021.08.015, PMID: 34509695

[ref18] Geerlings S. E. (2016). Clinical presentations and epidemiology of urinary tract infections. Microbiol Spectr 4. doi: 10.1128/microbiolspec.UTI-0002-2012 27780014

[ref19] Guo Y. Yao J. Sun C. Wen Z. Wang X. (2016). Characterization of the Deep-Sea Streptomyces sp. SCSIO 02999 derived Vap C/VapB toxin-antitoxin system in *Escherichia coli*. Toxins (Basel) 8:195. doi: 10.3390/toxins8070195, PMID: 27376329 PMC4963828

[ref20] Gupta K. Tripathi A. Sahu A. Varadarajan R. (2017). Contribution of the chromosomal ccdAB operon to bacterial drug tolerance. J. Bacteriol. 199:e00397-17. doi: 10.1128/jb.00397-17, PMID: 28674066 PMC5585702

[ref21] Harding G. K. Ronald A. R. (1994). The management of urinary infections: what have we learned in the past decade? Int. J. Antimicrob. Agents 4, 83–88. doi: 10.1016/0924-8579(94)90038-8 18611593

[ref22] Harms A. Brodersen D. E. Mitarai N. Gerdes K. (2018). Toxins, targets, and triggers: an overview of toxin-antitoxin biology. Mol. Cell 70, 768–784. doi: 10.1016/j.molcel.2018.01.003 29398446

[ref23] Hirakawa H. Suzue K. Kurabayashi K. Tomita H. (2019). The Tol-pal system of Uropathogenic *Escherichia coli* is responsible for optimal internalization into and aggregation within bladder epithelial cells, colonization of the urinary tract of mice, and bacterial motility. Front. Microbiol. 10:1827. doi: 10.3389/fmicb.2019.01827, PMID: 31456768 PMC6698795

[ref24] Infante A. Ortiz de la Tabla V. Martín C. Gázquez G. Buñuel F. (2021). Rapid identification and antimicrobial susceptibility testing of gram-negative rod on positive blood cultures using Micro scan panels. Eur. J. Clin. Microbiol. Infect. Dis. 40, 151–157. doi: 10.1007/s10096-020-04014-3, PMID: 32860091

[ref25] Jung C. Brubaker L. (2019). The etiology and management of recurrent urinary tract infections in postmenopausal women. Climacteric 22, 242–249. doi: 10.1080/13697137.2018.1551871, PMID: 30624087 PMC6629580

[ref26] Lecointre G. Rachdi L. Darlu P. Denamur E. (1998). *Escherichia coli* molecular phylogeny using the incongruence length difference test. Mol. Biol. Evol. 15, 1685–1695. doi: 10.1093/oxfordjournals.molbev.a025895, PMID: 9866203

[ref27] LeRoux M. Laub M. T. (2022). Toxin-antitoxin systems as phage defense elements. Ann. Rev. Microbiol. 76, 21–43. doi: 10.1146/annurev-micro-020722-013730, PMID: 35395167

[ref28] Levin B. R. Rozen D. E. (2006). Non-inherited antibiotic resistance. Nat. Rev. Microbiol. 4, 556–562. doi: 10.1038/nrmicro1445, PMID: 16778840

[ref29] Li M. Gong L. Cheng F. Yu H. Zhao D. Wang R.. (2021). Toxin-antitoxin RNA pairs safeguard CRISPR-Cas systems. Science 372:eabe5601. doi: 10.1126/science.abe5601, PMID: 33926924

[ref30] Li Y. Liu X. Tang K. Wang W. Guo Y. Wang X. (2020). Prophage encoding toxin/antitoxin system PfiT/PfiA inhibits Pf4 production in *Pseudomonas aeruginosa*. Microb. Biotechnol. 13, 1132–1144. doi: 10.1111/1751-7915.13570, PMID: 32246813 PMC7264888

[ref31] Liu S. Wu N. Zhang S. Yuan Y. Zhang W. Zhang Y. (2017). Variable Persister gene interactions with (p)pp Gpp for Persister formation in *Escherichia coli*. Front. Microbiol. 8:1795. doi: 10.3389/fmicb.2017.01795, PMID: 28979246 PMC5611423

[ref32] Ma D. Mandell J. B. Donegan N. P. Cheung A. L. Ma W. Rothenberger S.. (2019). The toxin-antitoxin Maz EF drives *Staphylococcus aureus* biofilm formation, antibiotic tolerance, and chronic infection. MBio 10:e01658-19. doi: 10.1128/mBio.01658-19, PMID: 31772059 PMC6879715

[ref33] Massot M. Daubié A. S. Clermont O. Jauréguy F. Couffignal C. Dahbi G.. (2016). Phylogenetic, virulence and antibiotic resistance characteristics of commensal strain populations of *Escherichia coli* from community subjects in the Paris area in 2010 and evolution over 30 years. Microbiology (Reading) 162, 642–650. doi: 10.1099/mic.0.000242, PMID: 26822436 PMC6365622

[ref34] McLellan L. K. Hunstad D. A. (2016). Urinary tract infection: pathogenesis and outlook. Trends Mol. Med. 22, 946–957. doi: 10.1016/j.molmed.2016.09.003, PMID: 27692880 PMC5159206

[ref35] Melican K. Sandoval R. M. Kader A. Josefsson L. Tanner G. A. Molitoris B. A.. (2011). Uropathogenic *Escherichia coli* P and type 1 fimbriae act in synergy in a living host to facilitate renal colonization leading to nephron obstruction. PLoS Pathog. 7:e1001298. doi: 10.1371/journal.ppat.1001298, PMID: 21383970 PMC3044688

[ref36] Micenková L. Bosák J. Štaudová B. Kohoutová D. Čejková D. Woznicová V.. (2016). Microcin determinants are associated with B2 phylogroup of human fecal *Escherichia coli* isolates. Microbiology 5, 490–498. doi: 10.1002/mbo3.345, PMID: 26987297 PMC4906000

[ref37] Monack D. M. (2013). Helicobacter and salmonella persistent infection strategies. Cold Spring Harb. Perspect. Med. 3:a010348. doi: 10.1101/cshperspect.a010348, PMID: 24296347 PMC3839601

[ref38] Nasrollahian S. Halaji M. Hosseini A. Teimourian M. Armaki M. T. Rajabnia M.. (2022). Genetic diversity, Carbapenem resistance genes, and biofilm formation in UPEC isolated from patients with catheter-associated urinary tract infection in north of Iran. Int. J. Clin. Pract. 2022, 9520362–9520311. doi: 10.1155/2022/9520362, PMID: 36187911 PMC9507725

[ref39] Portman D. J. Gass M. L. (2014). Genitourinary syndrome of menopause: new terminology for vulvovaginal atrophy from the International Society for the Study of Women’s sexual health and The North American Menopause Society. Climacteric 17, 557–563. doi: 10.3109/13697137.2014.946279, PMID: 25153131

[ref40] Qiu J. Zhai Y. Wei M. Zheng C. Jiao X. (2022). Toxin-antitoxin systems: classification, biological roles, and applications. Microbiol. Res. 264:127159. doi: 10.1016/j.micres.2022.127159, PMID: 35969944

[ref41] Ramisetty B. C. Santhosh R. S. (2016). Horizontal gene transfer of chromosomal type II toxin-antitoxin systems of *Escherichia coli*. FEMS Microbiol. Lett. 363:fnv238. doi: 10.1093/femsle/fnv238, PMID: 26667220

[ref42] Rycroft J. A. Gollan B. Grabe G. J. Hall A. Cheverton A. M. Larrouy-Maumus G.. (2018). Activity of acetyltransferase toxins involved in Salmonella persister formation during macrophage infection. Nat. Commun. 9:1993. doi: 10.1038/s41467-018-04472-6, PMID: 29777131 PMC5959882

[ref9002] Sambrook J. Russell D. W. (2001). Molecular cloning: a laboratory manual. 3. New York: Cold Spring Harbor Laboratory Press.

[ref43] Schumacher M. A. Balani P. Min J. Chinnam N. B. Hansen S. Vulić M.. (2015). Hip BA-promoter structures reveal the basis of heritable multidrug tolerance. Nature 524, 59–64. doi: 10.1038/nature14662, PMID: 26222023 PMC7502270

[ref44] Shah D. Zhang Z. Khodursky A. Kaldalu N. Kurg K. Lewis K. (2006). Persisters: a distinct physiological state of *E. coli*. BMC Microbiol. 6:53. doi: 10.1186/1471-2180-6-53, PMID: 16768798 PMC1557402

[ref45] Sonika S. Singh S. Mishra S. Verma S. (2023). Toxin-antitoxin systems in bacterial pathogenesis. Heliyon 9:e14220. doi: 10.1016/j.heliyon.2023.e14220, PMID: 37101643 PMC10123168

[ref46] Sycz Z. Wojnicz D. Tichaczek-Goska D. (2022). Does secondary plant metabolite Ursolic acid exhibit antibacterial activity against Uropathogenic *Escherichia coli* living in single- and multispecies biofilms? Pharmaceutics 14:1691. doi: 10.3390/pharmaceutics14081691, PMID: 36015317 PMC9415239

[ref47] Tartof S. Y. Solberg O. D. Manges A. R. Riley L. W. (2005). Analysis of a uropathogenic *Escherichia coli* clonal group by multilocus sequence typing. J. Clin. Microbiol. 43, 5860–5864. doi: 10.1128/jcm.43.12.5860-5864.2005, PMID: 16333067 PMC1317175

[ref48] Vandervelde A. Drobnak I. Hadži S. Sterckx Y. G. Welte T. De Greve H.. (2017). Molecular mechanism governing ratio-dependent transcription regulation in the ccdAB operon. Nucleic Acids Res. 45, 2937–2950. doi: 10.1093/nar/gkx108, PMID: 28334797 PMC5389731

[ref9003] Wang D. Dorosky R. J. Han C. S. Lo C. C. Dichosa A. E. Chain P. S. (2015). Adaptation genomics of a small-colony variant in a Pseudomonas chlororaphis 30-84 biofilm. Appl. Environ. Microbiol. 81, 890–899. doi: 10.1128/aem.02617-1 25416762 PMC4292466

[ref49] Wang X. Lord D. M. Cheng H. Y. Osbourne D. O. Hong S. H. Sanchez-Torres V.. (2012). A new type V toxin-antitoxin system where mRNA for toxin GhoT is cleaved by antitoxin GhoS. Nat. Chem. Biol. 8, 855–861. doi: 10.1038/nchembio.1062, PMID: 22941047 PMC3514572

[ref50] Wei Q. Ma L. Z. (2013). Biofilm matrix and its regulation in *Pseudomonas aeruginosa*. Int. J. Mol. Sci. 14, 20983–21005. doi: 10.3390/ijms141020983, PMID: 24145749 PMC3821654

[ref51] White A. P. Sibley K. A. Sibley C. D. Wasmuth J. D. Schaefer R. Surette M. G.. (2011). Intergenic sequence comparison of *Escherichia coli* isolates reveals lifestyle adaptations but not host specificity. Appl. Environ. Microbiol. 77, 7620–7632. doi: 10.1128/aem.05909-11, PMID: 21908635 PMC3209187

[ref52] Wood T. L. Wood T. K. (2016). The HigB/HigA toxin/antitoxin system of *Pseudomonas aeruginosa* influences the virulence factors pyochelin, pyocyanin, and biofilm formation. Microbiology 5, 499–511. doi: 10.1002/mbo3.346, PMID: 26987441 PMC4906001

[ref53] Xu J. Xia K. Li P. Qian C. Li Y. Liang X. (2020). Functional investigation of the chromosomal ccdAB and hipAB operon in *Escherichia coli* Nissle 1917. Appl. Microbiol. Biotechnol. 104, 6731–6747. doi: 10.1007/s00253-020-10733-6, PMID: 32535695 PMC7293176

[ref54] Yamaguchi Y. Park J. H. Inouye M. (2011). Toxin-antitoxin systems in bacteria and archaea. Annu. Rev. Genet. 45, 61–79. doi: 10.1146/annurev-genet-110410-132412 22060041

[ref55] Yu X. Gao X. Zhu K. Yin H. Mao X. Wojdyla J. A.. (2020). Characterization of a toxin-antitoxin system in *Mycobacterium tuberculosis* suggests neutralization by phosphorylation as the antitoxicity mechanism. Commun Biol 3:216. doi: 10.1038/s42003-020-0941-1, PMID: 32382148 PMC7205606

